# Is Cannabis Use Associated With the Worst Inpatient Outcomes in Attention Deficit Hyperactivity Disorder Adolescents?

**DOI:** 10.7759/cureus.2033

**Published:** 2018-01-07

**Authors:** Rikinkumar S Patel, Priya Patel, Kaushal Shah, Mandeep Kaur, Zeeshan Mansuri, Ramkrishna Makani

**Affiliations:** 1 Department of Psychiatry, Northwell Zucker Hillside Hospital; 2 Psychiatry, Windsor University School of Medicine; 3 Public Health, Western Kentucky University; 4 American University of Antigua; 5 Psychiatry, Texas Tech University Health Sciences Center at Odessa/permian Basin; 6 Child and Adolescent Psychiatry, Children’s Hospital of Philadelphia

**Keywords:** cannabis, marijuana, adhd, adolescent

## Abstract

Objective

To determine the impact of cannabis use disorder (CUD) on the inpatient outcomes of attention deficit hyperactivity disorder (ADHD) in adolescents

Background

Previous studies have evaluated the impact of CUD on the health-related quality of life in ADHD patients.

Methods

We used the nationwide inpatient sample (NIS) from the Healthcare Cost and Utilization Project (HCUP) from years 2010­–2014. We identified ADHD and cannabis use (CU) as the primary and the other diagnosis, respectively, using validated International Classification of Diseases, 9th Revision, and Clinical Modification (ICD­9–­CM) codes. We used the binomial logistic regression model to generate adjusted odds ratios (aOR).

Results

We analyzed a total of 11,232 ADHD adolescent hospital admissions from years 2010–2014; of these, 1.79% had CUD. The mean age of adolescents was 14.1 years (SD = 1.79). The prevalence of CUD was highest in ADHD adolescents of 15-18 years (73%) and common in the white race (71%). A higher proportion of ADHD with CUD was transferred to acute care hospitals and skilled/other nursing facilities (5.4% and 7.4% vs. 1.1% and 2.6%, respectively, p-value < 0.001). CUD increases the risk of inpatient charges > $12,247 (median) by 0.6 times (aOR = 1.835; p-value = 0.002) and increases the risk of inpatient stay > 5 days (median) by 0.7 times (aOR = 2.099; p-value < 0.001). The utilization of psychotropic medications was reduced by 0.8 times in ADHD with CUD adolescents by 0.8 times (aOR = 0.448; p-value = 0.017), and the implication of behavioral therapy in the management of ADHD with CUD adolescents was reduced by 0.9 times (aOR = 0.412; p-value = 0.048). Also, there is a 2.8 times higher risk of comorbid alcohol abuse in ADHD with CUD adolescents (aOR = 17.141; p-value < 0.001).

Conclusion

The increased risk of substance use is a long-term implication of ADHD in adolescents. It has been determined that comorbid CUD in patients with ADHD not only increases the risk of acute inpatient care but also prolongs the inpatient stay, thus increasing the healthcare cost. Surprisingly, comorbid CUD decreases the utilization of psychotropic medications and behavioral therapy in ADHD. Another major issue is the higher risk of comorbid alcohol abuse in ADHD with CUD adolescents. Further exploration with randomized controlled studies would be required to support and highlight the growing issue of cannabis use among adolescents with ADHD.

## Introduction

Cannabis is the most commonly used illicit drug for individuals aged 12 and over. Moreover, the prevalence of cannabis use in this age group has markedly increased from 6.2% in 2002 to 8.3% in 2015 [1]. As per the drug abuse warning network (DAWN), there were 456,000 cannabis-related emergencies in the United States in 2011, comparatively higher than the number of emergency department (ED) visits in 2009. Nonetheless, approximately 65% of these ED patients were male and 13% were in the age group of 12-17 years [2]. In light of this, further work should be focused on the identification of contributory factors causing an exacerbation of the substance use problem.

Attention deficit hyperactivity disorder (ADHD) is a common psychiatric disorder in childhood and adolescence that is characterized by impaired function in the areas of attention, hyperactivity, and impulsivity. ADHD and cannabis use disorders (CUD; i.e. cannabis abuse or dependence) often have clinical co-occurrence [3]. Earlier studies have suggested that cannabis may be a favorable substance among individuals with ADHD [4]. On the contrary, the results of another meta-analysis of the association of ADHD and CUD portrayed high levels of heterogeneity [5], suggesting that although a correlation appears to be present, single studies were highly influential to the meta-analytic results. Provided that extant findings generally support the CUD-ADHD association, several features of this association require further attention in order to delineate the unique role of inattention and hyperactivity-impulsivity symptoms in risk for CUD. The risk of substance use disorder was increased further with females with ADHD as opposed to males with ADHD in childhood [6].

The estimation of the population size of the increasing prevalence of ADHD [7] and cannabis use would be advantageous for the determination of optimal screening practices and policy intervention guidance. Previous studies focused on the prevalence and risk of association of CUD with ADHD, whereas other studies drew attention to the effect on the executive functioning and severity of hyperactivity/inattention symptoms in ADHD patients with CUD.

The primary objectives of this study are to analyze and discern the variance in the demographic pattern, risk of morbidity and mortality, disposition, inpatient length of stay, inpatient total charges, and utilization of psychotropic medications in ADHD patients in contrast to ADHD patients with CUD. To effectively assess the patient care of CUD in ADHD, the results obtained from this study will allow for strategic developments and clinical care models to screen and treat CUD and improve the utilization of psychotropic medications in ADHD patients, together with the overall improvement of the patients’ quality of life by means of decreasing morbidity.

## Materials and methods

Data source

A retrospective analysis was performed using the healthcare cost and utilization project's (HCUP) nationwide inpatient sample (NIS) data from the years 2010 to 2014 [8]. The agency for healthcare research and quality (AHRQ) sponsors the HCUP databases that are specifically designed to determine and identify patterns in hospital utilization and cost across the United States. The HCUP-NIS database is the largest inpatient database available in the United States, which represents a sample of non-federal United States community hospitals. In 2010, there was an increase of 1,051 hospitals to 4,411 hospitals from 45 states in the United States that participated in NIS projects. A sample estimate of over 95% of discharges from the hospitals participated in NIS. To minimize the margin of error and represent all 50 states across the United States, we weighed the estimated samples. The large sample size available via the database facilitated further recognition and analyses of rare conditions, uncommon treatments, and special patient populations. To protect the privacy of individual patients, physicians, and hospitals, the state and hospital identifiers are de-identified. There are many clinical and non-clinical hospitalization data elements recorded in the HCUP NIS database. Sample non-clinical information is patient’s demographic data, hospital characteristics, and total charges. Sample clinical-related information includes primary and other diagnosis, procedure codes (includes ICD-9 codes), discharge status, and length of stay [8].

Variables of interest

Based on the ICD-9-CM diagnosis codes, we identified the individuals with a primary diagnosis of ADHD at the time of admission. Correspondingly, based on the ICD-9-CM diagnosis codes, the patients with a primary diagnosis of ADHD and another diagnosis as CUD patients at the time of admission had been identified as the comparison group. In HCUP databases, more than 14,000 ICD-9-CM diagnosis codes and 3,900 procedure codes had been mentioned. ADHD was identified using diagnosis code 314.00 and 314.01, and CUD was identified using diagnosis codes 304.30, 304.31, 304.32, and 305.2. The age limit was set between 12 years to 18 years to study the impact of CUD in ADHD adolescents.

To measure the differences in hospitalization outcomes in ADHD patients versus ADHD with CUD patients, the outcome variables of interest included the severity of illness that measures the loss of body functions, the risk of mortality that measures the likelihood of dying, the disposition of the patient, and in-hospital mortality. In the NIS, we defined death as in-hospital mortality and, in this paper, it is reported as all-cause. We calculated the length of inpatient stay as the number of nights the patient remained in the hospital for a particular discharge. The length of inpatient stay in this analysis was all-cause. Inpatient charges during hospitalization do not include professional fees and non-covered charges. If the source provided total inpatient charges with professional fees, the professional fees were removed from the charge during HCUP processing. For the analysis, among the predictor variables, transfers out of the current hospital setting, the total number of ICD-9 CM procedures, primary ICD-9 procedures, and the duration of the hospital stays were considered important. The utilization of psychotropic medications during inpatient stay was identified using ICD-9 CM procedure codes 94.25. And, the utilization of behavioral therapy during inpatient stay was identified using ICD-9 CM procedure codes 94.33, 94.39, 94.42, and 94.44.

Comorbidities are considered coexisting conditions to ADHD, which is the primary disorder under this study. AHRQ comorbidity software was utilized to generate binary variables. Using ICD-9-CM codes, this variable identified three comorbidities in the records of discharge alcohol abuse (291.0-291.3, 291.5, 291.8, 291.81, 281.82, 291.89, 291.9, 303.00-303.93, 305.00-305.03), psychosis (295.00-298.9, 299.10, 299.11), and depression (300.4, 301.12, 309.00, 309.1, 311).

Approaches

A retrospective analysis was performed over the HCUP-NIS [8] database, focusing on the determination of the hospital outcomes for ADHD and ADHD with CUD patients. Descriptive statistics were used to summarize the results. The mean and standard deviations were used to explain the continuous variables. Pearson’s chi-square test and independent sample T-test were used for categorical data and continuous data, respectively. On the other hand, the categorical variables were shown in percentage values. We used a multinomial logistic regression model to measure the risk of associations between both groups in terms of inpatient length of stay (adjusted Odds Ratio (aOR)), inpatient total charges (aOR), and utilization of psychotropic medications (aOR). We applied discharge weights in all regression models, and the age, gender, race, median income of the patient’s zip code, and comorbidities were approximated. We used discharge weight, which is given in the NIS database to obtain nationally representative inpatient data. A p-value < 0.05 was used as a reference to determine the statistical significance test result. All statistical analysis was done by SPSS 23 (IBM, Armonk, New York, US) in this study [9]. Our study database does not contain patient identification. Thus, we were not required to take Institution Review Board (IRB) permission for this study.

## Results

Demographics

We analyzed 11,232 inpatient admissions for ADHD from 2010–2014 in adolescents and about 1.79% patients had comorbid CUD (N = 201). The mean age of ADHD patients at the time of hospitalization was 14.13 years (S.D. = 1.790, p-value < 0.001) and 15.89 years (S.D. = 1.458, p-value < 0.001) for ADHD with CUD patients. About 39.9% of ADHD patients and 85.1% of ADHD with CUD patients were in the age group of 15 to 18 years, comprising the most common age of presentation of ADHD and ADHD with CUD (p-value < 0.001) in adolescents.

ADHD and ADHD with CUD were found in the greater proportion of males (72.4% and 90.0%, respectively) (p-value < 0.001). Male gender is an important risk factor for CUD, as ADHD with CUD was seen in 90% males and only 10% females.

ADHD was discovered in 51.6% of whites whereas ADHD with CUD was found in 71.1% of whites. This indicates that the white race is an important risk factor for both ADHD and ADHD with CUD. A greater proportion of ADHD was found in blacks (27.2%) and Hispanics (16.1%) as compared to ADHD with CUD adolescents (16.4% and 8.6%, respectively) (p-value < 0.001). On the contrary, a greater proportion of ADHD with CUD was found in Asian or Pacific Islanders (3.9%) than in ADHD without CUD adolescents (0.6%) (p-value < 0.001).

The demographic distribution of the sample population is mentioned in Table 1.

**Table 1:** Distribution of ADHD and ADHD with CUD adolescents by demographics Significant p-values ≤ 0.05 at 95% confidence interval ADHD: attention deficit hyperactivity disorder; CUD: cannabis use disorder

Variable	ADHD	ADHD with CUD	p-value
Age (in years)			
Mean Age ± S.D.	14.13 ± 1.790	15.89 ± 1.458	< 0.001
12	24.7%	5.0%	< 0.001
13	19.1%	0%	
14	16.3%	10.0%	
15	14.8%	19.9%	
16	12.9%	29.4%	
17	8.1%	23.4%	
18	4.1%	12.4%	
Sex			
Male	72.4%	90.0%	< 0.001
Female	27.6%	10.0%	
Race			
White	51.6%	71.1%	< 0.001
Black	27.2%	16.4%	
Hispanic	16.1%	8.6%	
Asian or Pacific Islander	0.6%	3.9%	
Native American	0.5%	0%	
Other	4.0%	0%	
Median Household Income			
0-25th percentile	37.7%	26.0%	< 0.001
26th to 50th percentile	26.3%	12.5%	
51st to 75th percentile	20.3%	27.0%	
76th to 100th percentile	15.8%	34.5%	

Inpatient outcomes

A higher proportion of ADHD with CUD (97.5%) adolescents had non-elective or emergency admission compared to ADHD without CUD adolescents (86.1%) (p-value < 0.001). About 6.3% ADHD patients had a major loss of body function or severe morbidity compared to 5% ADHD with CUD patients (p-value < 0.001), whereas a moderate likelihood of dying was seen in a higher proportion of ADHD with CUD patients (2.5%) than in ADHD patients (0.7%) (p-value < 0.001).

The mean length of inpatient stay for ADHD with CUD adolescents was 4.81 days, which was lower as compared to ADHD without CUD adolescents who had a mean length of inpatient stay of 7.46 days (p-value = 0.003). The median length of stay for all patients was five days. But still, ADHD with CUD adolescents had a greater risk of longer inpatient stay of more than five days than ADHD without CUD adolescents (adjusted OR (aOR) = 2.099; 95% CI = 1.387 – 3.178; p-value < 0.001).

Despite a shorter duration of hospitalization, the mean inpatient total charges was higher in ADHD with CUD adolescents (USD 22,602) compared to ADHD without CUD adolescents (USD 18,398) (p-value < 0.001). The median inpatient total charges for all patients was USD 12,247. ADHD with CUD adolescents had a greater risk of higher inpatient charges of more than USD 12,247 than ADHD patients (aOR = 1.835; 95% CI = 1.244 – 2.705; p-value = 0.002). The association of the utilization of psychotropic medications was 0.8 times lower in ADHD with CUD adolescents compared to ADHD adolescents without CUD (aOR = 0.448; 95% CI = 0.232 – 0.864; p-value = 0.017). Also, the association of implication behavioral therapies in the management of ADHD is 0.9 times lower in adolescents with comorbid CUD (aOR = 0.412; 95% CI = 0.171 – 0.993; p-value = 0.048). 

A major proportion of ADHD with CUD patients (7.4%) was transferred to a skilled nursing facility (SNF), an intermediate nursing facility (INF), or another type of facility compared to 2.6% ADHD patients (p-value < 0.001). Also, 5.4% ADHD with CUD patients were disposed to short-term or acute care hospitals compared to 1.1% ADHD patients (p-value < 0.001). This indicates that greater numbers of ADHD with CUD patients needed specialized care in comparison to ADHD patients.

The distribution of the sample population based on inpatient outcomes is mentioned in Table 2, and the risk of adverse inpatient outcomes is mentioned in Table 3.

**Table 2:** Distribution of ADHD and ADHD with Cannabis Use adolescents by the inpatient outcomes Significant p-values ≤ 0.05 at 95% confidence interval ADHD: Attention Deficit Hyperactivity Disorder; CU: Cannabis Use Disorder; SNF: Skilled nursing facility; INF: Intermediate nursing facility; USD: United States Dollars

Inpatient Outcomes	ADHD	ADHD with CUD	p-value
Admission Type			
Non-elective	86.1%	97.5%	< 0.001
Elective	13.9%	2.5%	< 0.001
Severity of Illness			
Minor loss of Body Function	36.3%	44.3%	< 0.001
Moderate Loss of Body Function	57.3%	50.7%	< 0.001
Major Loss of Body Function	6.3%	5.0%	< 0.001
Risk of Mortality			
Minor Likelihood of Dying	99.2%	97.5%	0.019
Moderate Likelihood of Dying	0.7%	2.5%	
Comorbidities			
Alcohol Abuse	4.1%	43.3%	< 0.001
Depression	18.0%	14.9%	0.043
Psychosis	34.4%	27.5%	< 0.001
Inpatient Outcomes			
Mean Length of Inpatient Stay (days)	7.46	4.81	0.003
Mean Inpatient Charges (USD)	18,398	22,602	< 0.001
Disposition / Transfer			
Routine	95.4%	84.7%	< 0.001
Short-term Hospital	1.1%	5.4%	< 0.001
SNF, ICF, or Other Facility	2.6%	7.4%	< 0.001
Home Health Care	0.6%	2.5%	< 0.001
Against Medical Advice	0.4%	0%	< 0.001

**Table 3:** Risk of adverse inpatient outcomes in ADHD with CUD adolescents Significant p-values ≤ 0.05 at 95% confidence interval ADHD: attention deficit hyperactivity disorder; CUD: cannabis use disorder; USD: United States Dollars; aOR: adjusted odds ratio; CI: confidence interval

Inpatient Outcome in ADHD with CUD	aOR	95% CI	p-value
Length of Stay > 5 days	2.099	1.387 – 3.178	< 0.001
Inpatient Charge > USD 12,247	1.835	1.244 – 2.705	0.002
Utilization of Psychotropic Medications	0.448	0.232 – 0.864	0.017
Utilization of Behavioral Therapy	0.412	0.171 – 0.993	0.048

Comorbidities

Comorbid alcohol abuse is seen in 43.3% ADHD with CUD adolescents, which is 10 times higher than seen in ADHD without CUD adolescents (p-value < 0.001). Thus, there is a 2.8 times higher risk of comorbid alcohol abuse in ADHD with CUD adolescents (aOR = 17.141; 95% CI = 11.748 – 25.010; p-value < 0.001). On the contrary, comorbid depression and psychosis is seen in a lower proportion of ADHD with CUD adolescents compared to those without CUD (14.9% vs. 18.0%; p-value = 0.043 and 27.5% vs. 34.4%; p-value < 0.001, respectively). The risk of comorbidities in ADHD with CUD adolescents is mentioned in Table 4.

**Table 4:** Risk of comorbidities in ADHD with CUD adolescents Significant p-values ≤ 0.05 at 95% confidence interval ADHD: attention deficit hyperactivity disorder; CUD: cannabis use disorder; aOR: adjusted odds ratio; CI: confidence interval

Comorbidities in ADHD with CUD	aOR	95% CI	p-value
Alcohol Abuse	17.141	11.748 – 25.010	< 0.001
Depression	0.338	0.175 – 0.653	< 0.001
Psychosis	0.628	0.423 – 0.933	0.021

## Discussion

This analysis of population-based hospital data from adolescents admitted with ADHD and ADHD with CUD discloses the impact of CUD on hospitalization and related outcomes in ADHD. Our study had analyzed the NIS data with hospitalizations from 2010 to 2014, and over a five-year period, 1.79% of ADHD adolescents had comorbid CUD. As per a previous study, the risk of substance use disorder is higher for females as opposed to males with ADHD [6]. But as per our study, the male gender is an important risk factor for CUD, as ADHD with CUD was seen in 90% males and only 10% females. Among the adolescents, about 85% of ADHD with CUD patients were in the 15 to 18 years' age group, which supports the fact that cannabis is the most preferred substance among adolescents with ADHD [1,4]. While ADHD was seen in 51.6% of whites, ADHD with comorbid CUD was present in about 71.1% of whites. So, the white race is an important risk factor for both ADHD and ADHD with CUD.

A study done by Dalsgaard et al. states that ADHD patients with CUD have a higher risk of inattention and hyperactivity-impulsivity symptoms [6]. As per our results, about 7.5% ADHD adolescents with comorbid CUD had a major loss of body function or severe morbidity compared to 7% ADHD adolescents (p-value < 0.001). Due to this, ADHD with comorbid CUD adolescents had higher mean inpatient charges (USD 22,602) despite a shorter duration of hospitalization. But patients with CUD have a 0.8 times lower probability of utilizing psychotropic medications as compared to ADHD adolescents without CUD. Also, there is a 0.9 times lower probability of including behavioral therapies in the management of ADHD in adolescents with CUD. So, there are very high chances of ADHD with CUD adolescents facing adverse discharge plans as a majority of these patients in comparison with ADHD without CUD patients were transferred to an SNF, INF, or another health facility (7.4%) or disposed to a short-term or acute care hospital (5.4%). There is 2.8 times higher risk of comorbid alcohol abuse in ADHD with CUD adolescents, and due to this, it is seen in 10 times greater proportion than in ADHD without CUD adolescents.

To point out, the noteworthy strength of our study lies in the national representation of the dataset with the inclusion of a uniform collection of data through ICD-9-CM codes over the span of five years. To our knowledge, this is the first study to report the impact of CUD in ADHD adolescents regarding hospital outcomes, morbidity, mortality, and the utilization of psychotropic medications and behavioral therapy. With the intention of obtaining an appropriate sample size, we included 11,232 ADHD adolescents and 201 ADHD adolescents with CUD through the incorporation of the NIS dataset. The NIS dataset is subject to a minimal reporting bias. In addition, all the information from this dataset is coded independently of the practitioner, which makes it a reliable source.

However, our study has several limitations. As an administrative database, the NIS dataset lacks the patient-level data needed to make accurate clinical associations. Furthermore, such retrospective studies are always subject to selection bias, which might be accentuated by the moderate sensitivity of diagnostic codes for ADHD and CUD. Given the nature of the database, we could not account for re-hospitalizations despite the fact that they add to the total inpatient burden. All things considered, these potential limitations, when compared overall, present the NIS database as an unparalleled population-based perspective on disease associations with systematic and temporal factors providing a rationale for further in-depth studies.

## Conclusions

The increased risk of substance use is a long-term implication of ADHD in adolescents. It has been determined that comorbid CUD in patients with ADHD not only increases the risk of acute inpatient care but also prolongs the inpatient stay, thus increasing the healthcare cost. Surprisingly, comorbid CUD decreases the utilization of psychotropic medications and behavioral therapy in ADHD. Another major issue is the higher risk of comorbid alcohol abuse in ADHD with CUD adolescents. Further exploration with randomized controlled studies would be required to support and highlight the growing issue of cannabis use among adolescents with ADHD.
